# To Vaccinate or Not: The Relative Impact of Attitudes toward Autism Spectrum Disorders and the Ability to Interpret Scientific Information on Vaccination Decisions

**DOI:** 10.3390/ijerph17072542

**Published:** 2020-04-08

**Authors:** Einar B. Thorsteinsson, Anja Draper, Amy D. Lykins

**Affiliations:** School of Psychology, University of New England, Armidale, NSW 2351, Australia; anja.draper@gmail.com (A.D.); alykins@une.edu.au (A.D.L.)

**Keywords:** vaccine, scientific knowledge, attitudes, MMR, mumps, measles, rubella, autism spectrum disorder, ASD

## Abstract

Background. This pilot study investigated vaccine decision making, i.e., the relationships between knowledge and attitudes towards autism spectrum disorders (ASD), scientific literacy, attitudes toward the (MMR) vaccine, and children’s MMR vaccination status. Methods. A sample of 132 parents and expectant parents (mean age 38.40 years; >60% with university education) participated in a survey where they were asked about their knowledge of ASD, attitudes towards ASD and MMR, and their children’s MMR vaccine status. The participants also completed a standardized science test (The American College Test) to test their scientific literacy. Results. Knowledge of ASD was positively correlated with attitudes towards ASD. Attitudes towards ASD were positively correlated with scientific literacy and attitudes towards MMR. Attitudes towards MMR were positively correlated with MMR vaccine status (i.e., vaccination decision). Discussion. Factors other than scientific literacy seem to contribute towards children’s MMR vaccine status such as attitudes towards MMR. However, these are preliminary findings and need to be interpreted with caution.

## 1. Introduction

Autism Spectrum Disorder (ASD) is a complex neurodevelopmental disorder [1]. The disorder is characterized by persistent and pervasive deficits in social communication and social interactions across multiple situations. Deficits in social-emotional reciprocity are particularly evident in young children [2]. These characteristic symptoms typically present within the first two years of a child’s life [3], a developmental period which coincides directly with the peak time for administration of childhood vaccinations [4]. It is estimated that ASD currently affects 1 in every 160 individuals in the world today [5].

Studies concerning attitudes to ASD have primarily examined neurotypical children’s attitudes towards their peers diagnosed with ASD [6]. Public knowledge and understanding of ASD compared with other childhood disorders is considered to be poor [7,8] showing multiple misconceptions and a general lack of understanding and awareness of ASD [9]. Nonetheless, research aimed at investigating societal attitudes toward ASD is lacking [10], which is important, given the false proposition that ASD may be caused by the measles-mumps-rubella (MMR) vaccine. Thus, there is a need to examine the potential link between attitudes towards ASD and vaccines, especially the MMR vaccine, due to the erroneous claim that the MMR vaccine could be linked to children developing ASD.

Of all the childhood vaccines, the MMR vaccine has a uniquely controversial history [11]. In 1998, Andrew Wakefield et al. asserted a link between the MMR vaccine and the development of ASD in young children, thus suggesting a causative explanation for ASD [12]. Due to the ensuing lack of any evidence supporting a causal connection between the MMR vaccine and ASD, the Lancet officially retracted the article in January 2010. Despite flawed research methodology and a substantial and undisclosed conflict of interest on the part of Andrew Wakefield, media coverage concerning the autism-vaccine link significantly impacted public awareness [13]. The impact of media reports on public understanding is considerable considering that a substantial proportion of mothers attributed their knowledge of adverse vaccine side effects to media sources such as television [14].

Research has suggested that a strong link exists between parental attitudes to the MMR vaccine and vaccination behaviors [11,15]. Factors found to strongly influence parental decisions related to MMR vaccination include knowledge of diseases and of the MMR vaccine [16], general concerns about vaccine safety [17], and being a parent of a child diagnosed with ASD [18]. In particular, perception concerning the risk of ASD following vaccination with MMR has been found to directly impact attitudes towards the vaccine [16].

The Cultural Theory of Risk [19] offers a potential explanation concerning cultural beliefs that polarize anti-vaccination and pro-vaccination groups [20]. The theory presumes there is a psychological predisposition for: (1) individuals to uphold beliefs that fit with personal worldviews [20]; and (2) for group membership to be divided according to consensual risk perceptions [21]. Inherent in this theory is that culture comes before facts, subsequently shaping attitudes and consequent behavior. Thus, despite exposure to scientific information about the safety of vaccines, personal disagreement with the information being imparted by researchers continues [22].

Despite its potential importance with regard to decision-making about vaccinations, scientific literacy remains a largely unexplored variable in these decisions. Scientific literacy is defined as “the level of understanding of scientific and technological constructs needed to function as citizens in a modern industrial society” ([23]. Research by Allchin [24] emphasized that having an understanding about the nature of science provides individuals with fundamental skills necessary to identify credible sources of scientific findings, to understand value judgments applied to scientific findings, and to discern inaccurate information.

A study by Wallace, et al. [25] demonstrated that providing parents with evidence-based and up-to-date information about MMR vaccines—thereby improving scientific literacy—significantly improved their attitudes to the vaccine. Despite this, scientific facts alone may not be sufficient to modify perceptions of risk attributed to non-evidence-based theories [26]; greater knowledge does not always equate with unambiguous acceptance [27]. Furthermore, as the Cultural Theory of Risk suggests, people are generally primarily interested in accessing information that supports their own personal views rather than contradictory information, even that which has a sound scientific basis.

Thus, the aims of the present study were to examine associations between attitudes towards the MMR vaccine, scientific literacy, knowledge of and attitudes towards ASD, and vaccine related decisions. The present study also aimed to examine the information sources used by different groups of participants and potential reasons for participants not to vaccinate. The present study hypothesized: (a) a positive correlation between knowledge of ASD and attitudes towards ASD; (b) a positive correlation between attitudes towards ASD and the MMR vaccine; (c) a positive correlation between scientific literacy and parental vaccination decisions along with attitudes towards ASD and the MMR vaccine; and (d) that high scientific literacy would be related with positive MMR vaccine behavior (i.e., children more likely to be vaccinated).

## 2. Materials and Methods

### 2.1. Participants

The online survey was initiated by 246 participants. Of these, 114 respondents did not complete all of the psychological measures and were therefore deleted from further analysis (46.3% dropout rate). Thus, data for 132 participants (92% female, 8% male) were used for analysis. Participants ranged in age from 20 to 73 with a mean age of 38.40 years (*SD* = 10.17). Three participants were expecting children while the rest had children already. Highest level of education ranged from primary school (0.8%), to University postgraduate degree (22.7%), secondary school (13.6%), tertiary certificate (24.2%), and University undergraduate degree (38.6%). Based on postcode, 20 participants (15.2%) lived overseas while the rest lived in Australia. Of the 129 participants that indicated immunization status/intention, 97 (75.2%) had or were going to immunize their child/children. In respect to the measures of scientific literacy, sufficient data were obtained for only 45 participants (82% female, 18% male). Given the dropout rate, power varied for different analyses with analyses with small sample sizes having a smaller chance of reaching statistically significant findings. De-identified data are available through an open access data depository [28].

### 2.2. Measures

Participants were asked about their age, sex, occupation, residential postcode, highest level of education, if they had children (and if so how many), if they were expectant parents, if they had immunized their child/children (i.e., “Have all your children been immunized with the measles, mumps, rubella (MMR) vaccine?”; if not they were asked “Please state why you chose not to immunize.”) The answers from these two questions were combined into variables called MMR immunization status (i.e., positive, has or will immunize against MMR; negative, has not and will not immunize against MMR; and uncertain). Finally, participants were asked “What information did you access in making your decision regarding vaccinating your child with the MMR vaccine?”, with answer options such as “Friends”, “Family”, “Health Nurse”, “Pediatrician”, and “Website”.

The Societal Attitudes to Autism Scale (SATA) [29] is a 16-item scale recently developed to measure both explicit and implicit societal attitudes to ASD. Scores range from 16 to 64, with higher scores indicative of favorable attitudes. The SATA has demonstrated good construct validity and internal consistency. Importantly, the scale can discriminate between groups based on level of knowledge about ASD [29].

The Autism Knowledge Survey-Revised (AKS-R) [30,31] is a 20-item scale used to assess individual knowledge concerning ASD. The scale was originally developed by Stone [32]. The original version has demonstrated internal consistency and stability over time [33]. Scores range from 20 to 120, with higher scores indicative of greater knowledge.

The MMR Survey [34] is a 19-item scale developed to measure attitudes regarding decisions related to immunization with the MMR vaccine. Scores range from 19 to 76, with higher scores indicative of more favorable attitudes toward the MMR vaccine. The scale has demonstrated acceptable levels of internal consistency and test-retest reliability.

Because we were interested in the ability to reason scientifically rather than broad-based scientific knowledge, we used questions from The American College Test (ACT, 2011) to assess scientific literacy. The ACT is a standardized national university admissions examination commonly employed in the United States. It comprises tests in four subject areas: English, Mathematics, Reading and Science. Successful completion of the science test requires the ability to: (a) recognize and understand basic features and related concepts in regard to the information provided; (b) critically examine relationships between information provided and the conclusions or hypotheses which were developed; and (c) make generalizations based on the provided information and subsequent conclusions, learn new information, or make predictions. For the purposes of this study, only questions from the science subtest were used. This subtest has demonstrated good reliability and acceptable content validity. Scores range from 0 to 21, with higher scores indicating higher levels of scientific literacy.

### 2.3. Procedure

Ethics approval for the study was granted by the university’s Human Research Ethics Committee (HE13-281).Parents and expectant parents were recruited through flyers that were posted in community centers, schools, a university website for research participant recruitment, community notice boards, Facebook sites (including pro-vaccination and anti-vaccination groups, parenting groups, and mothering groups) and advertised in school newsletters. Participation was voluntary and, aside from research participation for currently enrolled psychology students, no incentives were offered for participation. Participants were provided with a brief outline of the study, which included an electronic link to access Qualtrics software (2013) online. Once they accessed the survey, they were provided with further information concerning the study and their participation. After consent was given online, participants completed a demographic questionnaire, followed by the ACT, the SATA, the MMR Survey, and the AKS-R. Due to the observed high rate of participant dropout three months following commencement of the study (60%, 93 surveys commenced, 37 completed), it was determined that reversing the order of measures was warranted. Subsequently, participants were presented with the SATA, AKS-R and MMR Survey, followed by the ACT.

### 2.4. Statistical Analysis

The data were analyzed using SPSS v25 (IBM, Armonk, NY, USA). No adjustments were made for multiple correlation coefficients in the Pearson’s correlation matrix, as we were only interested in the hypothesized relationships. One-tailed *p*-values are reported, as the hypothesized relationships between variables were directional.

## 3. Results

Examining MMR immunization status, of 132 participants, 104 (78.8%) participants indicated a positive attitude toward MMR immunization (i.e., has or will immunize against MMR), 21 (15.9%) indicated a negative attitude toward MMR immunization (i.e., has not and will not immunize against MMR), and 7 (5.3%) of participants gave inconclusive or ambiguous answers.

Table 1 shows the correlation between the key outcome variables. There was a strong correlation between attitudes towards MMR and MMR vaccine status. Knowledge of ASD was strongly and positively correlated with attitudes towards ASD, and scientific literacy had a strong positive correlation with attitudes towards ASD. There were only weak correlations between attitudes towards ASD and attitudes towards MMR, and scientific literacy had a low correlation with attitudes towards the MMR vaccine.

Analysis of qualitative data was conducted with respect to the information respondents self-reported accessing when making decisions regarding vaccinating their child with the MMR vaccine. The percentage of parents who had accessed information from general practitioners (GP), pediatricians, health nurses, health centers, friends, family, and websites, according to vaccination status of children, is presented in Table 2. Participants that were positive towards the MMR vaccine were more likely to have sought information from a GP than their counterparts that were negative towards the MMR vaccine, while the latter were more likely to have sought information from friends and web sites.

The question “Please state why you chose not to immunize” was answered by 31 participants. Some answers implied regret in relation to not having immunized their children, such as “ill-informed concerns,” while others indicated that participants would vaccinate later (e.g., “Not old enough yet. Will vaccinate (MMR) at 12 months.”). The answers were categorized into seven categories (see Table 3). The full list of answers is available in the de-identified data file via an open access data depository [28].

## 4. Discussion

This study intended to further current understanding of societal attitudes to ASD, attitudes to the MMR vaccine, and the impact of attitudes and scientific literacy on parental decisions regarding vaccinating their children with the MMR vaccine. The first hypothesis predicting that knowledge of ASD would be predictive of attitudes to ASD was supported. Results identified that knowledge of ASD accounted for a moderate proportion of the variance in parental attitudes to ASD. This positive correlation demonstrated that parents who had a better understanding and awareness of ASD also held more positive attitudes towards the disorder. Furthermore, as parents’ knowledge of ASD increased, so did their favorable attitudes to ASD. Given the high mean on the AKS-R (Table 1), it could be considered that respondents had a high level of knowledge of ASD in general, contradicting the findings by Holt and Christensen [9] that suggested that most people have a general lack of understanding concerning ASD. This finding may be explained by how participants were recruited, that is, coming from Facebook groups that were anti- and pro-vaccine, thus potentially more knowledgeable about aspects of ASD than the general public.

The second hypothesis proposing a relationship between attitudes to ASD and attitudes to the MMR vaccine was supported. The positive correlation found between these variables demonstrated that parents who held more favorable attitudes towards ASD were more likely to hold more favorable attitudes towards the MMR vaccine. This is in line with findings by Smith, Yarwood and Salisbury [16] who identified that fears related to the unfounded link between ASD and MMR vaccine were subsiding. Whilst their study did not specifically assess attitudes to ASD, they did acknowledge the influence of overall parental attitudes on vaccination decisions.

Based on the supposition that many individuals still believe MMR vaccine causes ASD, it was also expected that less favorable attitudes towards ASD and the MMR vaccine would be associated with non-vaccination of children with the MMR vaccine. This hypothesis was supported. Unsurprisingly, parents who had vaccinated their children with the MMR were more likely to have favorable attitudes to the vaccine. A difference in parental attitudes to MMR between those who had vaccinated and those who had not vaccinated their children has been noted in prior research [11]. As such, the current study suggests that attitudes to the MMR vaccine, regardless of negative or positive attitudes to ASD, are associated with the vaccination or non-vaccination of children. The probability of this assumption is further strengthened considering that no questions on the MMR Survey relate specifically to ASD. Whilst “risks” (e.g., concerned about the ingredients and unsure of safety of vaccines) associated with MMR were predominant reasons given for non-vaccination of children, as aforementioned, only one of the 21 parents who had a negative MMR vaccine status (not going to immunize) voiced concerns related specifically to ASD.

The third hypothesis predicting a positive relationship between scientific literacy and attitudes to ASD was supported, thereby providing further support for the suggestion that changes in attitude can be promoted through learning how to think and reason critically and scientifically [35].

The final hypothesis that scientific literacy would positively influence parental vaccination behaviors was not supported (see Table 1). Level of scientific literacy was not significantly related to vaccination behavior. This may be due to the limited number of participants that completed the scientific literacy test. It also may be that it is not scientific literacy that influences vaccination decisions, but rather motivated reasoning, given the different sources of information the two MMR vaccine status groups had (see Table 2). The majority of the “no” group relies on the websites option while the majority of the “yes” group relies on GPs and other actual medical professionals. It seems as if the “no” group is searching for confirmation to confirm their beliefs rather than trusting health care professionals.

Interestingly, it appears that a greater proportion of parents who had not vaccinated utilized primarily social and online sources of information rather than healthcare experts and workers. Analysis of these data suggests that parents may have been more likely to access information provided by others who hold similar views as themselves in respect to the vaccination of children with MMR. As such, these findings offer support for the Cultural Theory of Risk, which theorizes that there is a psychological predisposition for one to access information that fits with one’s own worldviews and group membership, dependent on cultural affiliation [20].

### Limitations

The sample size for some of the analyses was somewhat limited, potentially due to the cognitive effort required to complete the science tests. Most participants completed the psychological measures and demographics while many had limited success at tackling the science tests. However, the science tests are considered to be a measure of high school reasoning ability and participants mostly had high levels of education. As such, future research may need to consider offering incentives when participation involves increased cognitive effort on the part of participants. Participants were also sought from Facebook pages that were anti- and pro-vaccines, thus the sample may have had some very contrasting subsamples that a bigger study could look at in detail.

Attitudes and knowledge were self-reported and therefore were subject to social-desirability bias. Future studies may elect to utilize non-self-report methods of attitude assessment, such as Implicit Associations Test measures. Second, given the high scores on the AKS-R, respondents who self-selected to participate in this study may have had a vested interest in ASD, either through personal experience or education. Future studies may benefit from asking questions to ascertain qualitative information about participants’ personal experience with ASD. The questionnaire package was lengthy and this may have contributed to the high attrition rate. Furthermore, the questionnaire package also meant that some questions were not included that probably should have been included such as age of children, and how long ago the parents made their MMR decision. Future studies might also examine in more detail the intention vs. actual behavior of parents when it comes to vaccinating their children or not. Finally, given the research area, it is not known if the order of questions may have affected the answers to following questions, thus future studies may want to implement a random ordering of questions where possible. Finally, future studies might want to examine differences between males and females and thus target males to participate in the respective studies.

## 5. Conclusions

The findings from the present pilot study should be interpreted with caution. The results suggest that increasing public awareness and understanding of ASD may encourage more positive and favorable attitudes to the disorder. However, results obtained with respect to the current sample of respondents do not support the hypothesis that parental attitudes to ASD or the ability to interpret scientific information impact decisions related to the vaccination of children. Parental attitudes to the MMR vaccine itself, irrespective of attitudes to ASD, appeared to be the most significant factor determining vaccination behavior. Even if parents have not immunized it is important to ask why, as many are simply waiting for their child the reach the appropriate age for the given vaccination. It is also clear that the refusal to vaccinate is driven by several different reasons such as negative medical family experiences that are related to vaccinations by the parents, concerns about the safety of the vaccines and ingredients more specifically, and for religions or ethical reasons.

## Figures and Tables

**Table 1:** Pearson’s correlation (n) matrix for key variables.

Variable	1	2	3	4	5
1. Knowledge of ASD	-	0.32 ** (131)	0.17 (35)	0.06 (131)	−0.05 (124)
2. Attitude towards ASD		-	0.32 * (35)	0.19 * (132)	0.09 (125)
3. Scientific literacy			-	0.09 (35)	−0.09 (33)
4. Attitudes towards MMR				-	0.74 ** (125)
5. MMR vaccine status					-
*n*	131	132	35	132	125
*M*	90.09	56.55	12.97	53.74	1.83
*SD*	89.00	57.00	14.00	56.00	2.00
Range possible	20–120	16–64	0–21	19–76	1–2
Range actual	74–110	46–64	5–19	33–67	1–2

Note. ASD = Autism spectrum disorders; MMR = measles, mumps, rubella vaccine. MMR vaccine status: 1 = No, 2 = Yes. No adjustments are made for multiple correlation coefficients in the correlation matrix as only specific correlational relationships were of interest, but the total matrix is reported for completeness. * *p* < 0.05 (one-tailed). ** *p* < 0.01 (one-tailed).

**Table 2:** Frequency within MMR Vaccine status group of information sources accessed by parents and expectant parents.

MMR Vaccine Status (n)	GP	Pediatrician	Health Nurse	Health Centre	Friends	Family	Web Site
No (21)	10 (47.6%)	6 (28.6%)	3 (14.3%)	3 (14.3%)	11 (52.4%)	8 (38.1%)	14 (66.7%)
Yes (104)	74 (71.2%)	20 (19.2%)	28 (26.9%)	30 (28.8%)	19 (18.3%)	26 (25.0%)	9 (8.7%)
*p*	0.018	0.168	0.111	0.084	<0.001	0.111	<0.001

Note. MMR = measles, mumps, rubella vaccine; Attitudes towards MMR 1 = negative not vaccinating, 2 = positive vaccinating; GP = General practitioner (medical doctor). The *p* values reported are one-tailed.

**Table 3:** Categorization of answers to the open ended question “Please state why you chose not to immunize” (n = 31).

Answer	n	%
Will immunize when child is old enough	7	22.6
Negative personal, child, or family experience	9	29.0
Concerned about the ingredients	3	9.7
Unsure of safety of vaccines	8	25.8
Ethical or religious reasons	2	6.5
Ill informed (by own admission)	1	3.2
Do not want to answer	1	3.2
